# Case report: Ureteric bud intestinal-type adenocarcinoma involving the cervix was misdiagnosed as a large cervical fibroid

**DOI:** 10.3389/fmed.2024.1374653

**Published:** 2024-04-12

**Authors:** Li-li Zhang, Li Wang, Dan-ni Zhang, Jun-tong Wu, Yuan Liu, Yan-ping Wang

**Affiliations:** ^1^College of Chinese Medicine, Changchun University of Chinese Medicine, Changchun, Jilin, China; ^2^Department of Obstetrics and Gynecology, The 964th Hospital, Changchun, Jilin, China; ^3^Obstetrics and Gynecology Diagnosis and Treatment Center, The Affiliated Hospital, Changchun University of Chinese Medicine, Changchun, Jilin, China

**Keywords:** ureteric bud intestinal-type adenocarcinoma, cervical metastatic intestinal-type adenocarcinoma, large cervical fibroid, metastatic malignancy, surgical treatment

## Abstract

**Background:**

Malignant tumors of the ureteric bud are not common, and cervical involvement is even rarer. So far, there have been no such cases in the literature.

**Case summary:**

A 50-year-old woman developed intermittent light bleeding in the past 7 months and lower abdominal pain in the past 2 months. The human papillomavirus 16 (HPV) DNA, P16 chemical staining, thinPrep cytology test (TCT), and cervical and cervical canal tissue biopsy were all negative. Pelvic color Doppler ultrasound exhibited incomplete mediastinal uterus and heterogeneous echo from the cervical canal to the posterior wall of the cervix. Pelvic contrast-enhanced CT showed left cervical mass, left retroperitoneal mass, absence of the left kidney, and mediastinal uterus. An increase in human epididymal protein 4 (HE4) (133.6 pmol/L) was detected, while other tumor markers were at normal levels. Based on these examination results, a diagnosis of “cervical fibroids, left retroperitoneal mass, incomplete mediastinal uterus, left kidney deficiency”[SIC] was conducted, and expanded hysterectomy, right adnexectomy, and left retroperitoneal mass resection were performed. Through intraoperative rapid pathological diagnosis, postoperative pathological diagnosis combined with the re-evaluation of laboratory, and imaging and intraoperative examination results, the patient was diagnosed with ureteric bud intestinal-type adenocarcinoma involving the cervix. The patient has been tracked and followed up for approximately 11 months. She underwent six courses of chemotherapy. At present, the medication has been discontinued for 4 months, and there is no recurrence, metastasis, or deterioration of the tumor.

**Conclusion:**

For large masses of the cervix, it is feasible for the operation to be performed, improving the prognosis. There were a few limitations. A preoperative aspiration biopsy of masses was not performed to differentiate benign from malignant. Preoperative urography was not performed to clarify the function of the malformed urinary system structure. Partial cystectomy should be performed simultaneously with the resection of the ureteric bud for intestinal-type adenocarcinoma. In this case, a partial cystectomy was not performed, which can only be compensated with postoperative chemotherapy. Moreover, this patient did not undergo genetic screening, and it is currently unclear whether there are any genetic mutations associated with ureteric bud intestinal adenocarcinoma.

## 1 Introduction

**Figure 1:**
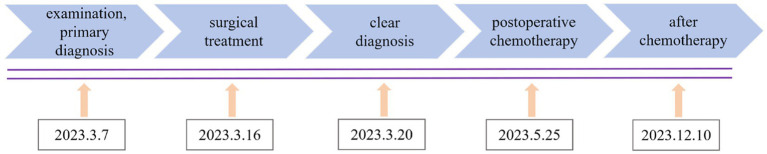
Timeline of historical and current episode of care.

Ureteric bud refers to the structure that protrudes from the mesonephric duct during the development of the kidney. The ureteric bud gradually evolves into the ureter, renal pelvis, renal calyx, collecting duct, and kidney through extension and repeated branching. Therefore, underdeveloped ureteric buds can lead to the abnormal development of the urinary system (1, 2). Due to the absence of obvious symptoms, it is usually discovered incidentally (3). Under long-term inflammatory stimulation and a hypoxic environment, the ureteric bud may undergo malignant transformation and involve the cervix (4). In this case, a 50-year-old woman sought medical attention because of irregular vaginal bleeding and lower abdominal pain. The examination revealed a large lump in the cervix, which was negative for cervical virology, cytology, and histopathology. She was initially diagnosed with cervical fibroids. After laboratory, imaging, intraoperative findings, and pathological reassessment, a definitive diagnosis of ureteric bud intestinal-type adenocarcinoma involving the cervix was determined (Figure 1).

## 2 Case presentation

**Figure 2:**
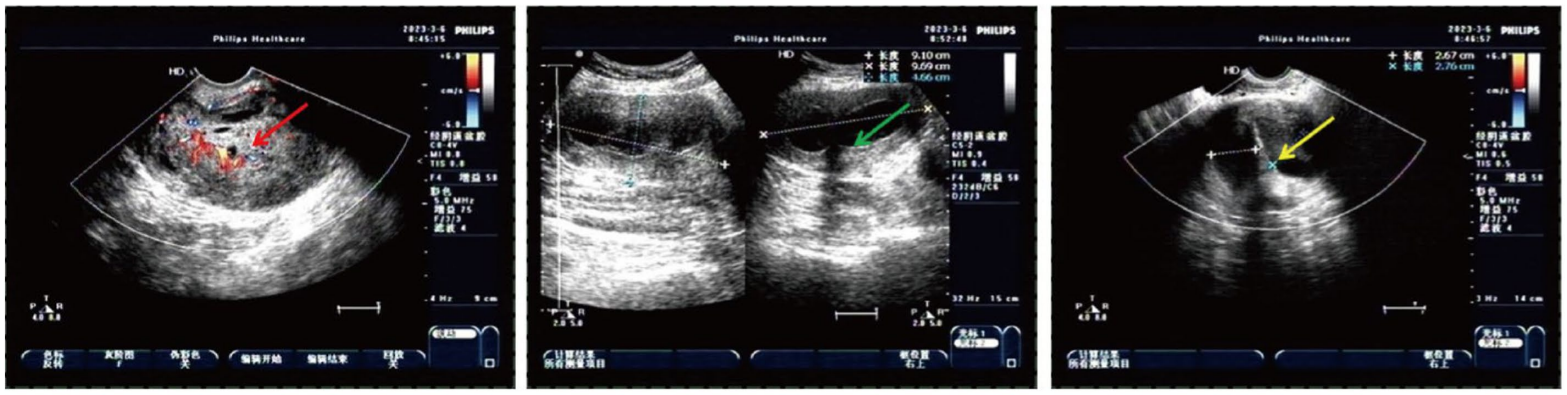
Heterogeneous echo on the left cervical wall and incomplete mediastinal uterus shown by pelvic ultrasound (The red arrow: heterogeneous echo on the left wall of the cervix detected with transvaginal ultrasound; the green arrow: heterogeneous echo on the left wall of the cervix detected with transabdominal ultrasound; and the yellow arrow: incomplete mediastinal uterus).

**Figure 3:**
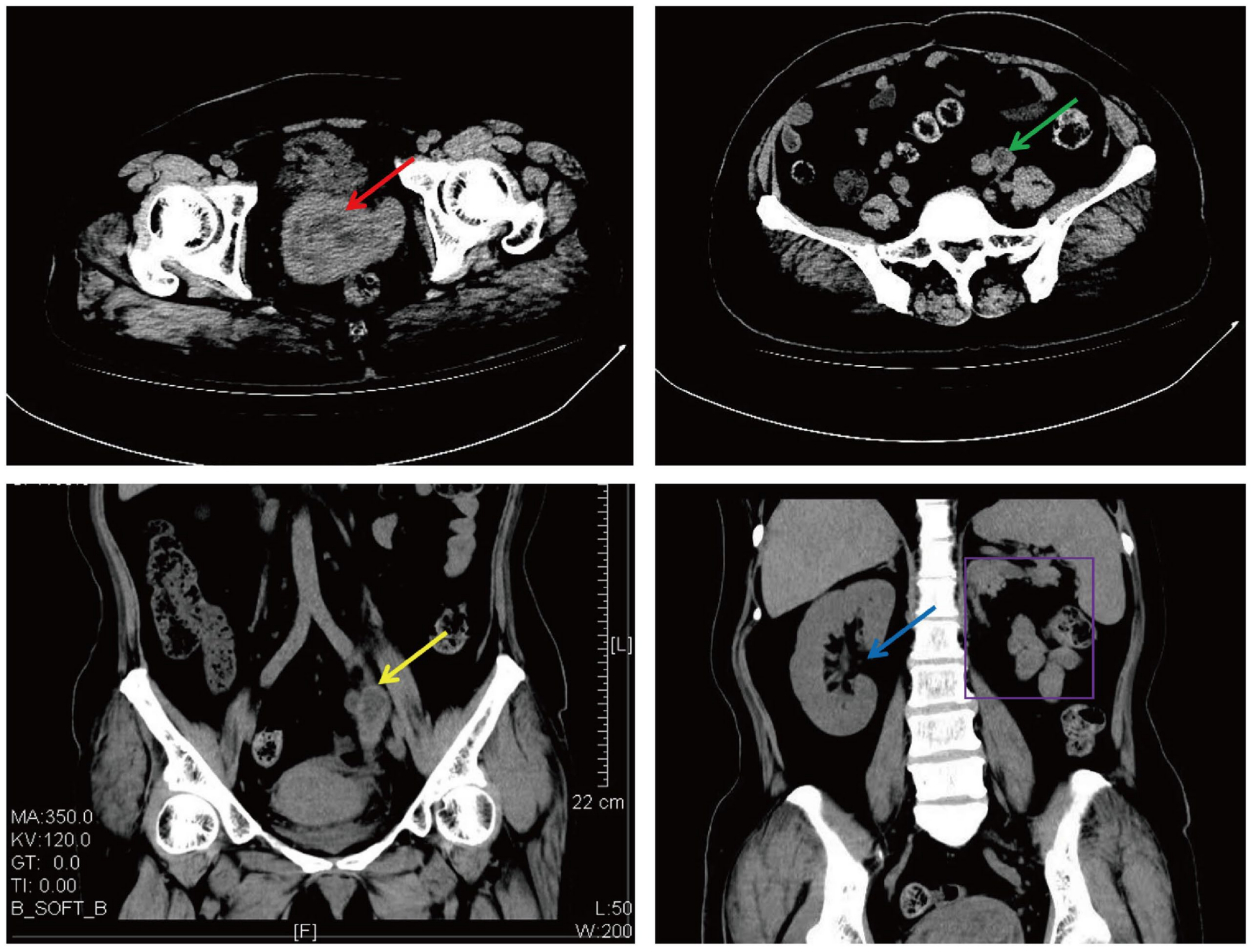
Cervical and left retroperitoneal mass shown on CT (The red arrow: a cervical mass; the green arrow: a horizontal left retroperitoneal mass; the yellow arrow: a coronal left retroperitoneal mass; the blue arrow: a coronal right kidney; and the purple border: a coronal left kidney absence).

A 50-year-old woman experienced intermittent irregular vaginal bleeding for 7 months and developed lower abdominal pain in the past 2 months. The patient had no history of pregnancy or childbirth, family genetic history, or adverse environmental exposure, and no bad habits such as smoking and drinking. Human papillomavirus 16 (HPV) DNA testing and p16 immunostaining were both negative. The ThinPrep cytology test (TCT) showed atypical hyperplasia of the glandular epithelium. Pathological examination of cervical and cervical canal scraping tissues suggested chronic cervicitis with squamous epithelial hyperplasia. Pelvic ultrasound indicated an incomplete mediastinal uterus and heterogeneous echo from the cervical canal to the posterior wall of the cervix, with a size of approximately 7.0×5.6×5.2 cm. The cervical serosal layer protrudes outward with irregular morphology and clear boundaries. Blood flow signals were observed inside and around the cervix (Figure 2). Pelvic contrast-enhanced CT showed that the mediastinum was seen in the uterus up to the top of the cervical os, and double uterine cavity changes were observed. A mixed-density mass was seen on the left side of the cervix with cystic low-density shadows and visible septa inside. The maximum cross-sectional size was approximately 7.7×7.8 cm, and cord-like high-density shadows were around it. The left kidney was absent. A solid cystic mass was visible on the left side of the retroperitoneum at the same level as the lower pole of the right kidney, extending downward to the left side of the cervix. The maximum cross-sectional size was approximately 3.4×2.4 cm, and the length was approximately 15 cm. The boundary with the cervix was not clear (Figure 3). A lump of approximately 7.0×6.0×5.0 cm, tender and inactivity, was palpated on the left side of the cervix during the gynecological examination. Tumor marker tests showed human epididymis protein 4 (HE4) at 133.6 pmol/L, with alpha-fetoprotein (AFP), carcinoembryonic antigen (CEA), carbohydrate protein 199 (CA199), carbohydrate protein 125 (CA125), human chorionic gonadotropin (HCG), carbohydrate antigen 153 (CA153), carbohydrate antigen 724 (CA724), risk of ovarian malignancy algorithm (Roman) index (premenopausal and postmenopausal), and squamous cell carcinoma (SCC) antigen all within the normal range. Preoperative diagnosis: (1) cervical fibroids; (2) left retroperitoneal mass; (3) incomplete mediastinal uterus; and (4) left kidney deficiency. Due to the increase in protein marker HE4, preoperative preparation was performed for malignant tumor surgery with adequate intestinal tract preparation. After full communication with the patient and family, we planned to perform a total hysterectomy and explore the retroperitoneal mass location.

During the operation, it was observed that the cervix was significantly enlarged to the low left, and a mass of approximately 7.0×6.0×5.0 cm protruded from the left wall of the cervix with a relatively clear boundary with surrounding tissues. It had a spherical shape, was isolated, and did not invade the rectum or bladder. The uterus, right ovary, and the complete large mass on the cervix were removed. The depth of the mass exceeded the external cervical os by 2 cm. The *ex vivo* specimen was cut open, and the tumor appeared to resemble smooth muscle tissue, with tough texture and multiple necrotic lesions. There was no tumor infiltration in the cervical canal. The results of rapid frozen pathology indicated a malignant cervical tumor. Intraoperative exploration revealed the other mass in the left retroperitoneum, approximately 15.0×3.0×2.0 cm in size, irregularly cylindrical in shape, with unclear boundaries with the surrounding area, especially closely adherent to the left cervical lesion, with gelatinous tissue on the surface. Intraoperative ultrasound examination showed that the mass lacked a complete renal pelvis structure, presented as a tubular cystic structure, and was interlinked but not connected to the bladder. The bladder was not involved, so a low-lying kidney was excluded. Based on preoperative imaging examination, intraoperative findings, and consultation with experienced urologists, this mass was considered an underdeveloped kidney and ureter, namely the ureteric bud. After explaining the situation to the patient’s family, an informed consent form was signed to perform an expanded hysterectomy, right adnexectomy, and retroperitoneal mass resection. The retroperitoneal mass specimen was cut open and showed a nodular shape with irregular wall thickness, a closed cavity filled with pus, necrotic tissue, and gel-like liquid.

**Figure 4:**
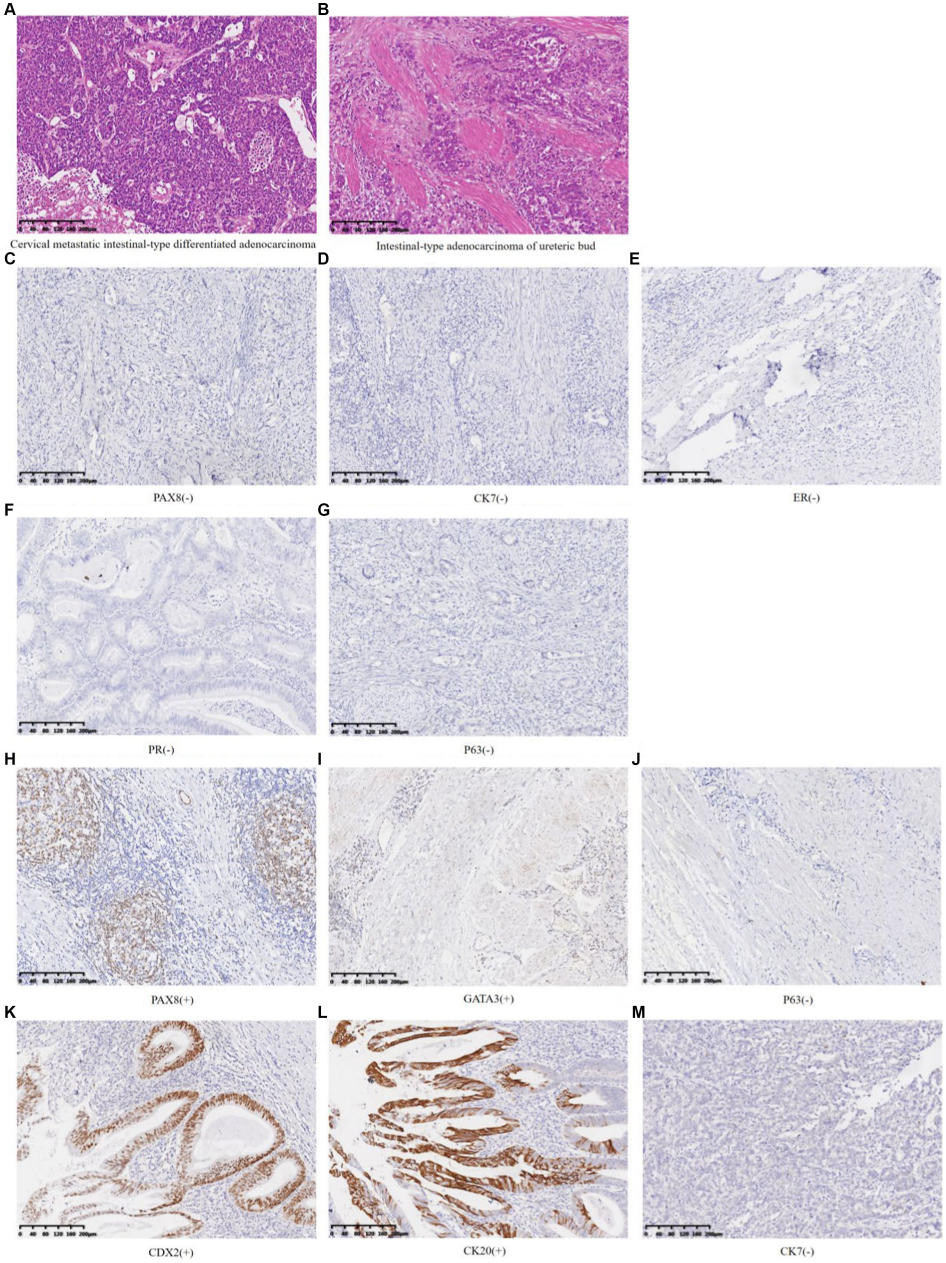
HE staining and immunohistochemistry of cervical masses and left retroperitoneal mass **(A)** Cervical metastatic intestinal-type differentiated adenocarcinoma (HE 10×). **(B)** Intestinal-type adenocarcinoma of the ureteric bud (HE 10×). **(C–G)** Cervical metastatic intestinal-type differentiated adenocarcinoma (IHC 10×). **(H–M)** Intestinal-type adenocarcinoma of the ureteric bud (IHC 10×).

The postoperative pathological diagnosis suggested cervical adenocarcinoma with intestinal-type differentiation in the total hysterectomy specimen. The tumor volume was 7.0 × 6.5 × 5.0 cm. The cancer cells were poorly differentiated, and solid cell clusters were often seen. The maximum diameter of the cancer cluster was 0.25 cm, with necrosis in the center, lymphocyte infiltration, and abscess formation around the cancer nest. Cervical serosa showed the diffuse infiltration of cancer cells. No tumor was found in the vasculature or nerves. The pathological results of the retroperitoneal mass showed a muscular tubular structure, with adenocarcinoma presented both inside and outside the tube wall (Figures 4A,B). The immunohistochemical (IHC) results showed that the cervical mass exhibited PAX8 (−), CK7(−), ER(−), PR(−), and P16(−) (Figures 4C–G). Retroperitoneal mass presented PAX8 focal(+), GATA3 focal(+), CDX2(+), CK20(+), CK7(−), and P63(−) (Figures 4H–M).

## 3 Discussion

Intestinal-type cervical adenocarcinoma usually presents as HPV16 (+). In this case, the HPV test was negative, and both TCT and cervical biopsy were negative, which was one of the factors leading to the misdiagnosis. In addition, this cervical metastatic mass was non-endophytic and non-exophytic on the cervical surface, but a large and isolated lesion occurred on the left wall of the cervix, becoming another factor for the misdiagnosis.

This case presented two cancerous lesions, a mass on the left cervical wall and a retroperitoneal mass. Whether they are primary lesions, secondary lesions, or two non-metastatic cancer lesions is the diagnostic challenge we need to focus on. PAX8 is highly sensitive and specific for diagnosing tumors of Müllerian origin, thyroid, and upper urinary/renal tract (5–7). GATA3 can serve as a biomarker for upper urinary tract tumors (8, 9). CDX2, CK20, and CK7 are of great significance in identifying whether malignant tumors are accompanied by intestinal-type differentiation (10–12). In this case, the IHC indicators of the cervical mass showed PAX8 (−) and CK7(−), indicating that this malignant tumor did not originate from the Müllerian duct. The IHC indicators of retroperitoneal mass, PAX8 and GATA3 expressed focal (+), along with the absence of transitional epithelial cells of the ureteral organ observed under the microscope and the absence of the left kidney and ureter, suggesting that during embryonic development, the ureteric bud may have developed into part of the kidney and ureteral tissue, which could undergo malignant transformation and manifest as PAX8 focal (+). However, most of these structures have not developed into normal ureteral and renal structures, and immunohistochemistry can show PAX8 (−). The entire structure can cause infection and carcinogenesis under long-term inflammation and hypoxia environment (13). Based on the preoperative cervical virus, shed cells, histopathological examination results, imaging, and intraoperative findings, especially immunohistochemical results, it was speculated that the malignant tumor of the ureteral bud was the primary lesion, and the cervical lesion was a secondary lesion. Non-metastatic tumors were excluded, and the immunohistochemical PAX8 (−) results of the cervical mass can be explained. Meanwhile, CDX2 (+), CK20 (+), and CK7 (−) demonstrated that this malignant tumor was accompanied by intestinal-type differentiation, ultimately leading to the diagnosis of ureteric bud intestinal-type adenocarcinoma involving the cervix. Nevertheless, there was not enough evidence to rule out the possibility that the cervix was the primary lesion and metastasized to the ureteric bud, causing similar adenocarcinoma. Based on imaging and intraoperative findings, the cervix tumor was considered at least stage IIB if it was the primary lesion. However, due to congenital left kidney deficiency, it was impossible to determine whether its function was missing and reach the diagnostic criteria of stage III.

The ureteric bud is a precursor structure that develops into the ureter and kidney (14). In the embryonic stage, due to genetic defects or genetic mutations, abnormal development of the ureteric bud leads to renal and ureteral underdevelopment, usually accompanied by ipsilateral renal hypoplasia or renal absence, ipsilateral bladder trigone underdevelopment, with residual ureteral blind segments of varying lengths, small or absent ureteral openings, or ureteral atresia, which is replaced by fibrous bands (15–17). Usually, those with congenital abnormalities of Müllerian duct development are often accompanied by abnormalities of the urogenital system (18, 19). In this case, the patient had an incomplete mediastinal uterus, accompanied by the absence of the left kidney. During the surgery, a tubular structure containing gel-like pus was seen below the same level as the right kidney. The location and shape were consistent with the imaging findings. This tubular object was considered to be an underdeveloped ureter, namely the ureteric bud. Its normal smooth muscle structure might be damaged by tumor tissue or infection, leading to tissue necrosis, or carcinogenesis accompanied by intestinal-type differentiation. This condition might affect adjacent organs when in a closed cystic cavity for a long time.

According to the international conventional standards for the diagnosis and treatment of cervical cancer, the size, location, and depth of the mass have exceeded the surgical scope, so radiotherapy and chemotherapy should be carried out directly. In this case, surgical treatment was prepared due to preoperative misdiagnosis as cervical fibroids, and the isolated cancer lesion with clear boundaries with surrounding tissues made it possible to completely remove the lesion through surgical resection. The patient has been tracked and followed up for approximately 11 months. After surgery, in an external hospital, the whole-body PET scan showed no mass lesions in other parts of the body, and routine chemotherapy was performed. The patient has completed six courses of chemotherapy (carboplatin AUC 5 plus Paclitaxel 135 mg/m^2^, every 21 days). The patient exhibited a high rate of adherence, and the chemotherapy process was uneventful, with no complications or serious adverse reactions. It cannot be ignored that during chemotherapy, the patient received health guidance and psychological support, which was beneficial for their recovery. At present, the medication has been discontinued for 4 months, and there is no recurrence, metastasis, or deterioration of the tumor. The patient is very satisfied with the overall treatment.

## 4 Conclusion

In summary, for large masses and isolated lesions on the lateral wall of the cervix, it is feasible for the operation to be performed by experienced doctors. The limitations of this study lie in the following points: First, preoperative aspiration biopsy of cervical masses and retroperitoneal masses was not performed to differentiate benign from malignant. Second, for patients with genitourinary malformations, especially those with confirmed mass by imaging and increased HE4 levels, preoperative urography was not performed to clarify the function of the malformed urinary system structure. Third, partial cystectomy should be performed simultaneously with the resection of the ureteric bud intestinal-type adenocarcinoma. In this case, a partial cystectomy was not performed, which can only be compensated with postoperative chemotherapy. Finally, this patient did not undergo genetic screening, and it is currently unclear whether there are any genetic mutations associated with ureteric bud intestinal adenocarcinoma.

## Data availability statement

The original contributions presented in the study are included in the article/supplementary material, further inquiries can be directed to the corresponding authors.

## Ethics statement

Written informed consent was obtained from the individual(s) for the publication of any potentially identifiable images or data included in this article. Written informed consent was obtained from the participant/patient(s) for the publication of this case report.

## Author contributions

L-lZ: Writing – original draft. LW: Writing – original draft. D-nZ: Writing – original draft. J-tW: Writing – review & editing. YL: Writing – review & editing. Y-pW: Funding acquisition, Writing – review & editing.
